# The nordic and baltic EMS data - quality indicators and benchmarking 2025

**DOI:** 10.1186/s13049-025-01488-y

**Published:** 2025-10-24

**Authors:** Tim A. Lindskou, Viljami Lampilinna

**Affiliations:** 1 Centre for Prehospital and Emergency Research at Danish Centre for Health Services Research, Aalborg University Hospital and Department of Clinical Medicine, Aalborg University, Aalborg, Denmark; 2 Finnish Institute for Health and Welfare, Helsinki, Finland

**Keywords:** Emergency medical services, Scandinavian and nordic countries, Ambulances, Baltic states, Quality indicators

## Abstract

**Background:**

In 2014 a prehospital emergency medical services (EMS) data co-operation began between the five Nordic countries, Sweden, Denmark, Norway, Finland, and Iceland. The Baltic State Estonia joined the group in 2020. The Nordic-Baltic EMS Network, that was formed to continue the project-based collaboration as a more permanent entity, has now published their 2025 report. This commentary aims to promote the report and its key results.

**Main body:**

The report describes the current population, healthcare organisation and EMS systems of each country. Similarities in the EMS systems was found between several of the countries, such as the use of electronic prehospital medical records, and variants of the Emergence Medical Coordination Centre decision support tool Norwegian Index for Emergency Medical Assistance. While many of the included countries have national quality indicators on EMS, a list of Nordic-Baltic EMS Quality and Benchmarking Indicators were defined. Availability of data for these *quality indicators* varied between each nation, yet it was possible to compare several. The quality indicators included *timepoints on dispatch and response*, *bystander CPR* and *return of spontaneous circulation*, *incidents*, *missions*, *patients advised or referred over the phone*, *non-conveyed patients*, *hospitalised patients*, and *transport missions*. There were discrepancies between the included countries, due to several issues. Data completeness, quality and interpretation is assessed as a major point. Others include legislation, access points for acute patients, such as general practitioners available out-of-hours, or other acute medical helplines. However, differences in general population and EMS patient population in terms of demography and disease pattern may also be present. These elements have not been analysed in depth in the current report and should be considered when interpreting and comparing the results.

**Short conclusion:**

Despite national differences, the Nordic-Baltic EMS Network report provide an overview of the EMS systems and quality indicators, enabling preliminary comparisons. The online report is intended to be updated on an annual basis when possible and has already led to the planning of a future indicators, project groups and research. Together this promotes international collaboration, and exchange of experiences.

## Background

In 2014 a prehospital emergency medical services (EMS) data co-operation began between the five Nordic countries, Sweden, Denmark, Norway, Finland, and Iceland. The Baltic State Estonia joined the group in 2020.

The objective of the Nordic-Baltic EMS Data project was initially to collect comparable data based on common quality indicators, and two previous reports have been published in 2019 and 2021 [1, 2]. Data primarily concerned ambulance missions and time points as only sparse patient data was available.

Since then, the objective has evolved to identify and develop common benchmarking as well as quality indicators of the EMS systems. Furthermore, to assess and recommend potential improvements and development of EMS.

The Nordic-Baltic EMS Network, that was formed to continue the project-based collaboration as a more permanent entity, has now published their 2025 report available as an online version and downloadable as PDF, at https://yhteistyotilat.fi/wiki08/display/JULNBDQ [3]. This commentary aims to promote the report and its key results.

## Main text

The report describes the current population and healthcare organisation. While previously described, the report also provides a up-to-date general description of each countries EMS system and presents a brief comparison via tables [4–7].

Many of the included countries have national quality indicators on EMS, however, to facilitate comparisons, a list of Nordic-Baltic EMS Quality and Benchmarking Indicators were defined. These relate to time points, number of calls, incidents and missions, and now also selected patient data, such as the main reason for contact with EMS and specific data on cardiac arrest.

Similarities in the EMS systems was found between several of the countries, such as the use of electronic prehospital medical records, and variants of the Emergence Medical Coordination Centre decision support tool Norwegian Index for Emergency Medical Assistance.

**Figure:**
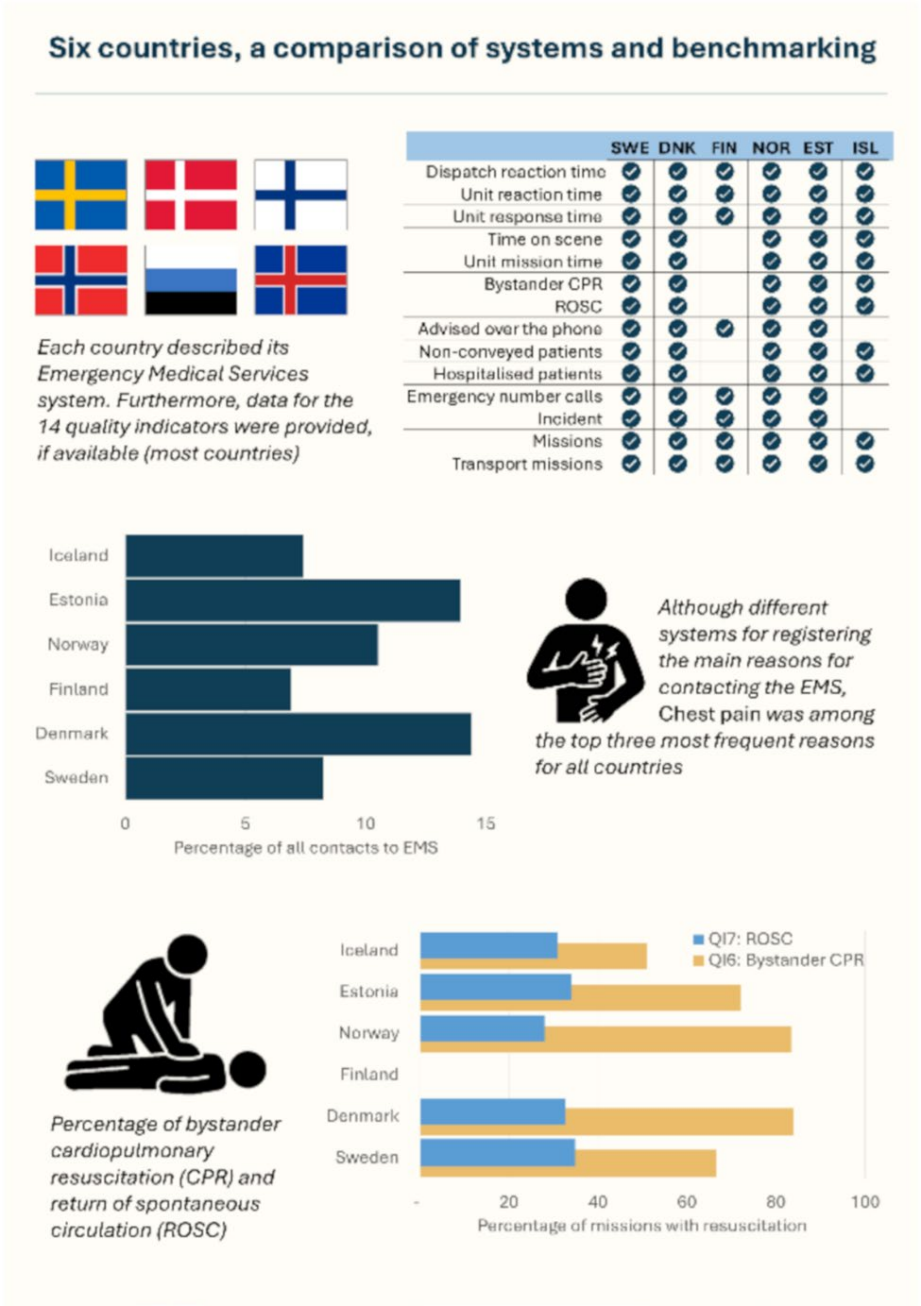

Availability of data for the *quality indicators* varied between each nation, yet it was possible to compare several. The quality indicators included *timepoints on dispatch and response, bystander CPR* and *return of spontaneous circulation, incidents, missions, patients advised or referred over the phone, non-conveyed patients, hospitalised patients*, and *transport missions*.

Sweden and Denmark had a higher proportion of hospitalised compared to non-conveyed patients (60/24, 40/15, and 66/17 per 100 capita respectively). On contrary, Norway and Estonia had a higher proportion of non-conveyed patients (25/21 and 112/21 per 100 capita respectively).

In cardiac arrest *bystander CPR* was slightly higher for Denmark and Norway (84 and 79 vs 51–72%) and return of spontaneous circulation for all countries were 0,07 - 0,20 per 1000 capita. Finally, *main reason for contact to EMS*, had chest pain among the top three reasons for all countries, accounting for 7 to 26 percent of all emergency calls. unclear problem was likewise high among most countries, ranging from 6 to 17 percent.

The cause for discrepancies between the included countries are multifaceted and described in more detail in the report. One contributor is data completeness, quality and interpretation. Furthermore, legislation, access points for acute patients, such as general practitioners available out-of-hours, or other acute medical helplines. Moreover, the discrepancies may reflect differences in general population and EMS patient population in terms of demography and disease pattern. Finally, differences in thresholds for calling for the EMS may also come into play. These elements have not been analysed in depth in the current report and should be considered when interpreting and comparing the results.

The Nordic-Baltic EMS Quality and Benchmarking Indicators gradually move away from being based on ambulance response times, to patient centered quality indicators based on electronic prehospital medical records. This patient data is absolutely needed when comparing patient populations and outcomes.

## Conclusions

Despite national differences, the Nordic-Baltic EMS Network report provide an overview of the EMS systems and quality indicators, enabling preliminary comparisons.

The online report is intended to be updated on an annual basis when possible, and promote exchange of knowledge as well as facilitate international collaboration, development and improvements in the Nordic-Baltic EMS Network systems.

## Data Availability

Not applicable.
